# Silencing of HuR Inhibits Osteosarcoma Cell Epithelial-Mesenchymal Transition *via* AGO2 in Association With Long Non-Coding RNA XIST

**DOI:** 10.3389/fonc.2021.601982

**Published:** 2021-03-19

**Authors:** Yongming Liu, Yuan Zhang, Jinxue Zhang, Jingchang Ma, Xuexue Xu, Yuling Wang, Ziqing Zhou, Dongxu Jiang, Shen Shen, Yong Ding, Yong Zhou, Ran Zhuang

**Affiliations:** ^1^ Orthopedic Department of Tangdu Hospital, The Fourth Military Medical University, Xi’an, China; ^2^ Institute of Medical Research, Northwestern Polytechnical University, Xi’an, China; ^3^ Department of Immunology, The Fourth Military Medical University, Xi’an, China

**Keywords:** osteosarcoma, epithelial-mesenchymal transition, migration, HuR, AGO2, long non-coding RNA XIST

## Abstract

**Background:**

Osteosarcoma (OS) is a highly malignant and aggressive bone tumor. This study was performed to explore the mechanisms of HuR (human antigen R) in the progression of OS.

**Methods:**

HuR expression levels in OS tissues and cells were detected by immunohistochemistry and western blotting. HuR siRNA was transfected into SJSA-1 OS cells to downregulate HuR expression, and then cell proliferation, migration, and epithelial-mesenchymal transition (EMT) were evaluated. RNA immunoprecipitation was performed to determine the association of the long non-coding RNA (lncRNA) XIST and argonaute RISC catalytic component (AGO) 2 with HuR. Fluorescence *in situ* hybridization analysis was performed to detect the expression of lncRNA XIST. Western blotting and immunofluorescence assays were performed to observe AGO2 expression after HuR or/and lncRNA XIST knockdown.

**Results:**

Knockdown of HuR repressed OS cell migration and EMT. AGO2 was identified as a target of HuR and silencing of HuR decreased AGO2 expression. The lncRNA XIST was associated with HuR-mediated AGO2 suppression. Moreover, knockdown of AGO2 significantly inhibited cell proliferation, migration, and EMT in OS.

**Conclusion:**

Our findings indicate that HuR knockdown suppresses OS cell EMT by regulating lncRNA XIST/AGO2 signaling.

## Introduction

Osteosarcoma (OS), which mainly occurs in children and young adults, is one of the most prevalent malignant cancers, has a low survival rate, and poor overall prognosis. Despite the introduction of treatments combining surgery, chemotherapy, and radiotherapy, the 5-year survival rate of patients with metastatic OS is 11–30% (1, 2). Thus, studies are needed to explore the underlying molecular mechanisms involved in OS carcinogenesis, progression, and metastasis to find out potential therapeutic targets.

RNA-binding protein HuR (human antigen R), also known as ELAVL1 (ELAV-like RNA-binding protein 1), binds to elements rich in adenylate and uridylate (AU-rich elements) to regulate the stability and translation of various mRNAs (3, 4). Because of its pivotal role in stabilizing the mRNA of key factors involved in carcinogenesis, the effects and therapeutic potential of HuR in cancer have been intensively investigated (5). HuR is overexpressed in various tumors, such as lung cancer, breast cancer, and brain tumors (6–10). Recent studies reported that HuR levels were significantly upregulated in OS tissues compared to that in normal adjacent tissues (11, 12). However, additional studies are required to fully understand the role of HuR in OS.

Long non-coding RNAs (lncRNAs) are a large class of non-protein-coding transcripts with over 200 nucleotides in length (13). lncRNAs contribute to the unrestricted proliferation and invasion of cancer cells (14, 15). lncRNA X-inactive specific transcript (XIST) has emerged as a key regulator of OS. Its expression is significantly increased in OS tissues and cell lines, which is associated with poor prognosis (16, 17). However, the detailed mechanisms of how lncRNA XIST modulates OS progression remain unclear.

Here, we determined the HuR expression level in OS tissues and cell lines. The effects of silencing of HuR on OS proliferation, migration, and epithelial-mesenchymal transition (EMT) processes were examined and its binding partners were predicted.

## Methods

### Clinical Samples

A tissue array of patients with malignant OS was purchased from the Alenabio Biological Technology Company (Xi’an, China). Clinical pathological data included age, sex, pathology diagnosis, and TNM grading, which were collected by assessing the medical records of patients with OS who had undergone surgery. No patients had been administered preoperative treatment or had co-occurrence of other diagnosed tumors.

### OS Cell Lines

Human OS cell lines were purchased from the American Type Culture Collection (Manassas, VA, USA), whereas the human normal osteoblast cell line hFOB1.19 was obtained from Jennio Biotech Co., Ltd. (Guangzhou, China). Cells were cultured in Dulbecco’s modified Eagle’s medium (Thermo Fisher Scientific, Waltham, MA, USA) supplemented with 10% fetal bovine serum (Life Technologies, Carlsbad, CA, USA) with 5% CO_2_ at 37°C in a humidified incubator. Non-transfected cells were used as a blank group and random small interfering RNA (siRNA)-transfected cells were used as the negative control group. HuR siRNA sequence: GAGGCAATTACCAGTTTCA, lncRNA XIST siRNA sequence: GCTGCAGCCATATTTCTTACT. AGO2 siRNA was obtained from Santa Cruz Biotechnology (Dallas, TX, USA; sc-44409). Lipofectamine™ 3000 (Invitrogen, Carlsbad, CA, USA) was used for cell transfection according to the manufacturer’s instructions. Commercially available lentiviral (LV)-HuR constructs (Tianyucheng Biotechnology, Xi’an, Shaanxi, China) were modified to overexpress HuR.

### Bioinformatics Analysis

Bioinformatic analysis (http://starbase.sysu.edu.cn/index.php) was performed to predict the potential target of HuR. The UALCAN database was used to analyze argonaute RISC catalytic component 2 (AGO2) gene expression across multiple cancer types (http://ualcan.path.uab.edu/index.html). UALCAN analysis of cancer data was based on The Cancer Genome Atlas database.

### Immunohistochemistry (IHC) Staining and Semi-Quantitative Analysis

We used a formalin-fixed paraffin-embedded tissue microarray for IHC analysis. The paraffin sections were deparaffinized, rehydrated, and treated with 3% hydrogen peroxide for 10 min. Antigen retrieval was performed in citrate solution (pH 6.0) in a steamer for 2 min. The sections were washed with phosphate-buffered saline (PBS) and incubated for 30 min in 5% bovine serum albumin. Anti-HuR primary antibody (Santa Cruz Biotechnology) and anti-AGO2 (Affinity Biosciences LTD, Jiangsu, China) were added and incubated overnight at 4°C. The sections were washed with PBS and incubated with horseradish peroxidase-conjugated goat anti-mouse secondary antibody for 30 min at room temperature. A complex of streptavidin-peroxidase and 3,3′-diaminobenzidine substrate was then applied for color development and the slides were counterstained with hematoxylin. A semi-quantitative score was generated based on the IHC staining intensity as described previously (18): +, weak staining; ++, moderate staining; +++, intense staining. The slides were photographed under an optical microscope (BX51, Olympus, Tokyo, Japan).

### Quantitative Real-Time PCR (qPCR) Assay

Total RNA was extracted from the cells using TRIzol™ Reagent (Thermo Fisher Scientific), and then reverse-transcribed into cDNA using SuperScript III Reverse Transcriptase from Invitrogen. PCR was performed using the SYBR Green Realtime PCR Master Mix (TAKARA, Shiga, Japan). Relative gene expression was quantified using the comparative Ct (2^−ΔΔCT^) method. β-actin served as an internal reference.

### Western Blotting

The cells were washed with cold PBS and lysed using RIPA lysis buffer (Beyotime Biotechnology, Haimen, China) supplemented with a protease inhibitor cocktail (Cell Signaling Technology, Danvers, MA, USA). A nuclear and cytoplasmic isolation kit was purchased from Beyotime Biotechnology and used according to the manufacturer’s instructions. Protein concentration was determined with the BCA protein assay kit (Thermo Fisher Scientific). Equal amounts of protein samples (30 μg) were subjected to SDS-PAGE, and then transferred onto polyvinylidene fluoride membranes (Millipore, Billerica, MA, USA). The membranes were blocked with 5% non-fat milk in Tris-buffered saline with 0.1% Tween 20 and blotted with corresponding primary antibodies at 4°C overnight. Proteins were blotted with horseradish peroxidase-conjugated secondary antibody (Cell Signaling Technology) and visualized with an enhanced ECL system (Tanon Science & Technology, Shanghai, China). β-actin was used as whole cell internal loading control. Lamin B1 and tubulin were employed as positive controls for the nuclear and cytoplasmic fractions, respectively.

### Cell Proliferation Analysis

Cell Counting Kit (CCK)-8 (Beyotime) was used to analyze cell proliferation after different treatments. The cells were seeded in triplicate into 96-well plates at 3,000 cells/well, followed by culturing for 24 h, 36 h, or 48 h. CCK-8 was added according to the manufacturer’s protocol. Absorbance was detected at 450 nm. The effect of HuR on OS cell proliferation was also evaluated in a 5-ethynyl-2′-deoxyuridine (EdU) incorporation assay (RiboBio, Guangzhou, China) according to the manufacturer’s instructions. Briefly, 5 × 10^3^ OS cells were plated in triplicate in 96-well plates and cultured for 48 h, followed by exposure to EdU for an additional 4 h at 37°C. The cells were fixed with 4% paraformaldehyde for 15 min and permeabilized with 0.5% Triton X-100 for 20 min at room temperature. After washing three times with PBS, the cells were reacted with Apollo reaction cocktail for 30 min. Nuclei were stained with 4’,6-diamidino-2-phenylindole (DAPI; Roche Diagnostics, Basel, Switzerland). EdU-positive cells were visualized under a fluorescent microscope (Olympus).

### Cell Cycle and Apoptosis Assay

Flow cytometry was performed to measure the cell cycle and apoptosis in the blank, NC siRNA, and HuR siRNA-transfected groups. After washing with PBS, the cells were fixed in 70% ethanol and precooled at 4°C. Propidium iodide was added to the cell precipitate according to the instructions of the DNA ploidy test kit (Sigma-Aldrich, St. Louis MO, USA). Cell apoptosis was measured using an Annexin V-fluorescein isothiocyanate and propidium iodide staining kit (Beyotime). The cells were evaluated by flow cytometry (Beckman Coulter, Brea, CA, USA). Each experiment was repeated at least three times. FlowJo software VX (FlowJo LLC, Ashland, OR, USA) was used to analyze the data. The degree of apoptosis was also determined using the One Step TUNEL Apoptosis Assay Kit (Beyotime Biotechnology) according to the manufacturer’s protocol. Cy3-positive cells were visualized under a fluorescent microscope (Olympus).

### Cell Scratch (Wound Healing Migration) and Transwell Migration Assay

OS cells were seeded into six-well plates and treated as described. After 48 h when the cells reached 100% confluence, a scratch was induced in the monolayer. Images of the wound area were captured immediately after the scratch (T0) and 12 h (T12) later to monitor cell migration into the wounded area, and the percentage of the scratch area (% scratch) closed was calculated as (width at T0 − width at T12)/width at T0 × 100. Images of the cells were obtained under an inverted microscope (CX41, Olympus). The experiment was performed three times. The migration assay was performed as described previously (19). A cell culture inserted with a polycarbonate membrane (8 μm pore size) was purchased from Corning Costar, Inc. (Corning, NY, USA).

### RNA Immunoprecipitation (RNA IP) Assay

OS cells were lysed with 25 mM of Tris-HCl buffer (pH 7.5) and RNase inhibitor (Sigma-Aldrich). After a pre-clearance procedure, the whole cell lysates were used for IP for 4 h using protein A agarose (Thermo Fisher Scientific) at room temperature in the presence of excess (30 μg) IP antibody (anti-HuR, control mouse IgG). RNA in the IP materials was extracted with TRIzol, followed by detection of *AGO2*, *lncRNA XIST*, and *β-actin* mRNA levels by qPCR. The experiment was repeated at least three times.

### Fluorescence *In Situ* Hybridization (FISH) Analysis of lncRNA

The cells were placed on glass chamber slides and cultured. The cells were first fixed with 4% paraformaldehyde in PBS for 30 min, and then permeabilized with 0.1% Triton X-100. Next, the cells were washed and treated with pre-hybridization buffer. The FISH detection kit including probes for lncRNA XIST and U6 was purchased from RiboBio Co., Ltd. (Guangzhou, China). Hybridization was carried out in a humidified chamber for 16 h, followed by staining with DAPI.

### Immunofluorescence Assay

The cells were fixed with 4% paraformaldehyde and permeabilized with 0.1% Triton X-100, followed by blocking with 5% bovine serum albumin for 30 min. The cells were incubated with anti-HuR (Santa Cruz Biotechnology) or anti-AGO2 antibodies (Abcam, Cambridge, UK) for 1 h at room temperature. After washing the cells, they were incubated with Cy3-labeled goat anti-mouse secondary antibody and fluorescein isothiocyanate-labeled goat anti-rabbit secondary antibody for 1 h at room temperature, and then stained with DAPI. The images were visualized under a Leica LSM 800 confocal immunofluorescence microscope (Wetzlar, Germany).

### Statistical Analysis

All data were statistically analyzed using THE GraphPad Prism version 6 software (La Jolla, CA, USA). All data are presented as means ± standard deviation. Data were analyzed using the independent sample *t*-test for comparison between two groups, and one-way ANOVA with Tukey multiple comparison test between three or more groups. Statistical analyses for clinical data were performed using SPSS (version 10.0; SPSS, Inc., Chicago, IL, USA). Pearson χ^2^ test was used to evaluate the statistical significance of the association between HuR expression and clinical features (n = 30). Statistical significance was defined as *P* < 0.05.

## Results

### HuR Is Overexpressed in OS Cells

**Figure 1:**
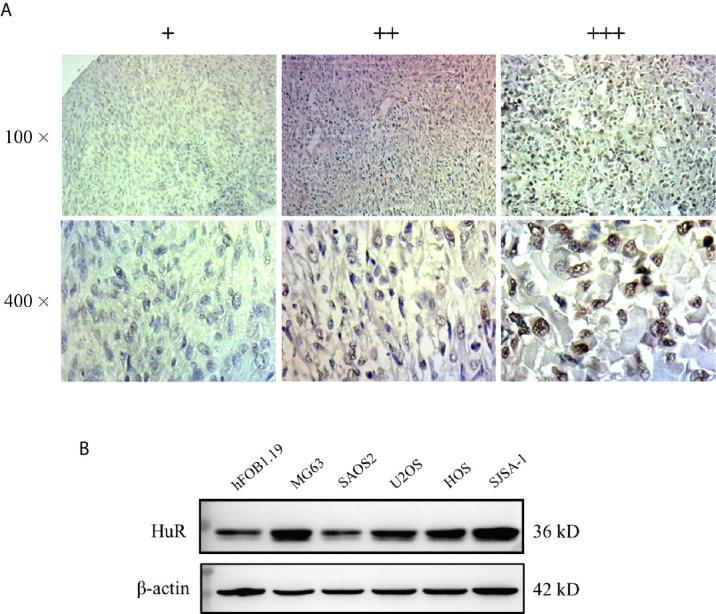
Expression of HuR protein in tissues from patients with osteosarcoma (OS) and in OS cells. **(A)** Representative images of different IHC staining intensities of HuR are shown in OS tissues. Staining patterns were categorized into three groups as follows: weak staining (+), moderate staining (++), and intense staining (+++). Upper panel, original magnification 100x; lower panel, original magnification 400x. **(B)** Western blotting analysis of HuR expression in a human osteoblast cell line (hFOB1.19) and OS cell lines (MG63, SAOS2, U2OS, HOS, and SJSA-1).

We measured the expression of HuR in different OS tissues and cell lines to evaluate the roles of HuR in OS formation and progression. First, we examined HuR expression in OS tissues by IHC staining. The results showed that HuR is mainly localized in the cell nucleus (**Figure 1A**). Comparisons between HuR expression and the clinicopathological characteristics of OS are shown in **Table 1**. It was shown that the HuR expression had no significant correlation with gender and age of OS patients. All 30 OS samples were in either the N0 stage, in which there was no tumor metastasis to nearby lymph nodes, or M0 stage, indicating the absence of distant organ metastasis. Notably, HuR expression was significantly higher in the T2 stage than that in the T1 stage of OS tumors (*P* = 0.001).

**Table 1:** Correlation between HuR and clinical features of patients with OS (n = 30).

Variable		No. of patients	HuR expression			*P*-values
			+	++	+++	
Gender	Male	20 (66.7)	8	9	3	0.885
	Female	10 (33.3)	4	5	1	
Age	>20	20 (66.7)	7	10	3	0.441
	<20	10 (33.3)	5	4	1	
T stage	T1	7 (23.3)	7	0	0	**0.001**
	T2	23 (76.7)	5	14	4	

P-values based on a χ^2^ test; bold, statistically significant (P < 0.05).

Next, HuR expression was determined in the OS cell lines MG63, SAOS2, U2OS, HOS, and SJSA-1, and normal hFOB1.19. According to our results, HuR was elevated in most OS cell lines, and MG63 and SJSA-1 cells showed a high level of HuR expression (**Figure 1B**). Consistent with the findings of recent studies (11, 12), our results further indicated that HuR is related to the tumor progression in OS.

### Silencing HuR Increases Sensitivity to Apoptosis of OS Cells

**Figure 2:**
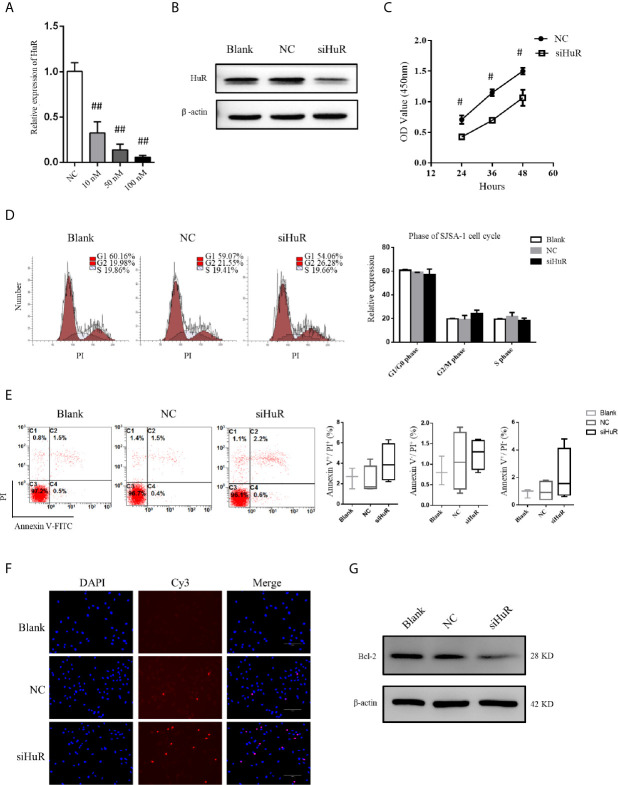
Effect of HuR knockdown on progression of OS cells. **(A)** qPCR analysis for HuR knockdown efficiency at 48 h after siRNA transfection in SJSA-1 OS cells. ^##^ indicates *P* < 0.01 compared to the scramble siRNA negative control (20). **(B)** Western blotting analysis of HuR at 48 h after siRNA transfection in SJSA-1 cells. β-actin was used as an internal control. **(C)** SJSA-1 cells with NC siRNA or HuR siRNA (siHuR) transfection were subjected to a CCK-8 assay to examine cell viability. ^#^ indicates *P* < 0.05 compared to the NC group. **(D)** Cell cycle regulation by HuR was detected by propidium iodide staining and flow cytometric analysis in SJSA-1 cells. **(E)** Cell apoptosis was detected by Annexin-V/PI staining and flow cytometric analysis in SJSA-1 cells. Results are representative of at least three independent experiments. **(F)** TUNEL staining in the SJSA-1 cells harvested from blank, negative control, and siHuR groups. Very few TUNEL-positive cells were detected in the blank and NC groups. Scale bar = 125 μm. **(G)** Representative image of western blotting for Bcl-2. β-actin served as an internal loading control.

qPCR and western blotting confirmed the knockdown efficiency. siRNA at a concentration of 50 nM was used to knockdown HuR in SJSA-1 cells (**Figures 2A, B**). Because HuR expression was significantly higher in the T2 stage than the T1 stage of the OS tumor, we first determined whether HuR promotes OS cell proliferation. CCK-8 assay was performed to analyze whether HuR expression affects cell proliferation (**Figure 2C**). Consistent with the results of Pan et al. (12), knockdown of HuR inhibited cell viability and proliferation. Additionally, the effects of HuR knockdown on cell cycle and apoptosis were examined by flow cytometric analysis. The assay showed a tendency that silencing of HuR decreased the SJSA-1 cells in G1/G0 and increased G2/M cell cycle progression of the cells, but with no significance (**Figure 2D**). No significant difference in apoptosis was observed between each group through flow cytometric analysis (**Figure 2E**). As shown in **Figure 2F**, there were slightly more TUNEL-positive OS cells in the siHuR group when compared with the blank and NC groups. Furthermore, there was a lower expression of Bcl-2, an apoptotic suppressor, in HuR siRNA-transfected OS cells (**Figure 2G**). Altogether, the mentioned results suggest that knocking down HuR could make OS cells more sensitive to apoptotic stimuli.

We further used a lentiviral construct to overexpress HuR. The results showed that the expression level of HuR was significantly higher in the HuR overexpression (OE HuR) group; whereas the expression levels in HuR siRNA-transfected OS cells were almost undetectable. These results showed that lentiviral infection was efficient. As shown in **Supplementary Figures 1A, B**, western blotting analysis for Bcl-2 and the TUNEL assay indicated that HuR overexpression decreased apoptosis level in OS cells. Besides, to further confirm the biological function of HuR overexpression in OS cells, a rescue experiment was performed with HuR siRNA in HuR overexpressed OS cells. The expression of HuR was significantly downregulated in the siRNA + OE group compared with OE HuR group. Meanwhile, Bcl-2 level and the TUNEL assay indicated that rescue experiments increased the sensitivity of apoptosis level compared with that in HuR overexpressed OS cells.

### Silencing of HuR Inhibites OS Cell Migration and EMT

**Figure 3:**
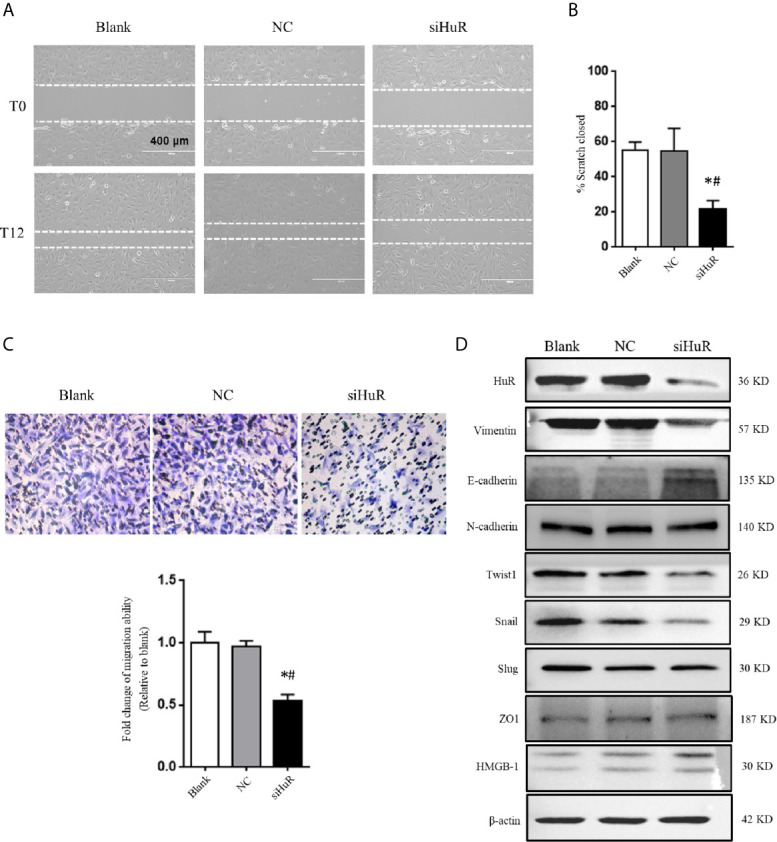
Silencing of HuR inhibits the migration and EMT of OS cells. **(A)** Photographs of cells in scratch assay. Untreated SJSA-1 cells (blank), their corresponding scramble siRNA (20), and HuR siRNA (siHuR) transfected cells were seeded into six-well plates. Images were obtained at 0 and 12 h after scratch to monitor cell migration for wound closure. **(B)** Statistical results of the scratch assay at 12 h. * indicates *P* < 0.05 compared to the blank group. ^#^ indicates *P* < 0.05 compared to the NC group. Representative images from three separate experiments are shown. **(C)** Representative images of transwell migration assay of SJSA-1 cells with HuR knockdown and respective blank control and NC siRNA. Lower panel shows the statistical graph of the transwell migration assay. * indicates *P* < 0.05 compared to the blank group. ^#^ indicates *P* < 0.05 compared to the NC siRNA. **(D)** Representative images of western blotting for EMT markers and related transcriptional factors. β-actin served as an internal loading control.

Next, wound healing and transwell migration assays were performed to detect the migration ability of SJSA-1 cells after different treatments. There was no difference in scratch closure in the NC-siRNA group compared to the blank group. However, there was a significant decrease in the percentage of scratch closure in cells lacking HuR compared to that in the NC-siRNA or blank group (**Figures 3A, B**). Additionally, the transwell migration assay revealed that HuR silencing reduced the number of migrated OS cells compared to that in the control groups (**Figure 3C**). The results showed that HuR knockdown significantly inhibited OS cell migration.

We next investigated the role of HuR knockdown in EMT of OS cells. The protein levels of the EMT markers vimentin, E-cadherin, and N-cadherin, as well as related key transcriptional factors (Twist1, Snail, and Slug) were examined by western blotting. The results showed that silencing of HuR decreased the levels of vimentin, Twist1, and Snail in SJSA-1 OS cells, while increased the level of E-cadherin. However, the levels of N-cadherin, Slug, HMGB-1, and ZO1 were not significantly changed after silencing HuR compared to the control groups in SJSA-1 cells (**Figure 3D**).

### HuR Binds to lncRNA XIST and Decreases Its Expression

**Figure 4:**
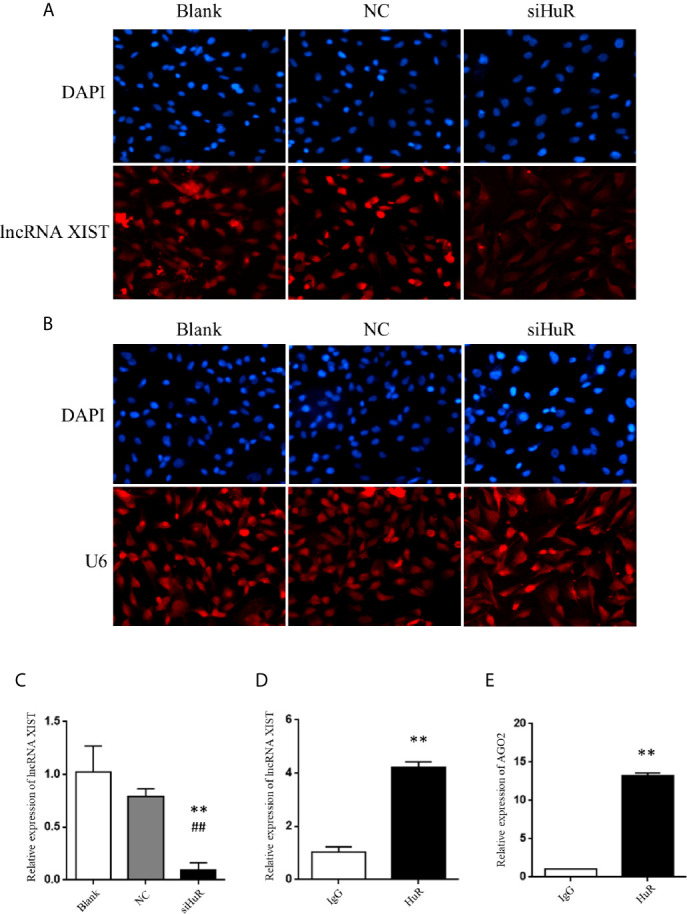
Silencing of HuR represses lncRNA XIST expression in OS cells. **(A)** RNA fluorescence *in situ* hybridization (FISH) analysis of lncRNA XIST with a specific probe in SASJ-1 OS cells. **(B)** U6, a ribonucleoprotein, served as the endogenous control for FISH analysis; the nucleus was stained with DAPI. **(C)** The mRNA level of lncRNA XIST was detected by qPCR in cells after different treatments. ** indicates *P* < 0.01 compared to the blank group. ^##^ indicates *P* < 0.01 compared to the NC group. qPCR was used to measure the lncRNA XIST **(D)** and AGO2 **(E)** abundance in the HuR-IP complex following the RIP assay. ** indicates *P* < 0.01 compared to the mouse IgG group.

We further explored the regulatory mechanisms involved in HuR-related OS cell EMT. Numerous studies have reported lncRNAs can interact with RNA-binding proteins in cancer. Recently, lncRNA XIST was demonstrated to promote the progression of OS (16, 21). To gain insight into the precise mechanism and potential interaction of HuR with XIST, we first examined its subcellular localization by FISH with or without HuR siRNA transfection. XIST was distributed in the both nucleus and cytoplasmic regions of OS cells, indicating that XIST acts as a regulatory RNA to influence target mRNAS. After silencing of HuR in OS cells, the level of XIST was downregulated (**Figure 4A**). U6, which is mainly localized in the cell nucleus, was used as an internal reference for staining, as observed in **Figure 4B**. qPCR analysis further demonstrated that lncRNA XIST expression was decreased in OS cells after HuR knockdown (**Figure 4C**). Additionally, the RNA IP assay revealed that lncRNA XIST was highly expressed in the RNA-protein complex that had been precipitated by the anti-HuR antibody (**Figure 4D**).

Considering that lncRNA-mRNA typically forms a regulatory network, we hypothesized that repression of XIST by HuR involved other factors associated with XIST. We searched starbase and predicted that both HuR and XIST could bind to AGO2, which is an integral component of the RNA-induced silencing complex (RISC). The results revealed high levels of AGO2 in the RNA-protein complex immune-precipitated by the anti-HuR antibody (**Figure 4E**). The results indicate that HuR binds to the lncRNA XIST and AGO2 in OS cells.

### AGO2 Is Regulated by HuR and lncRNA XIST

**Figure 5:**
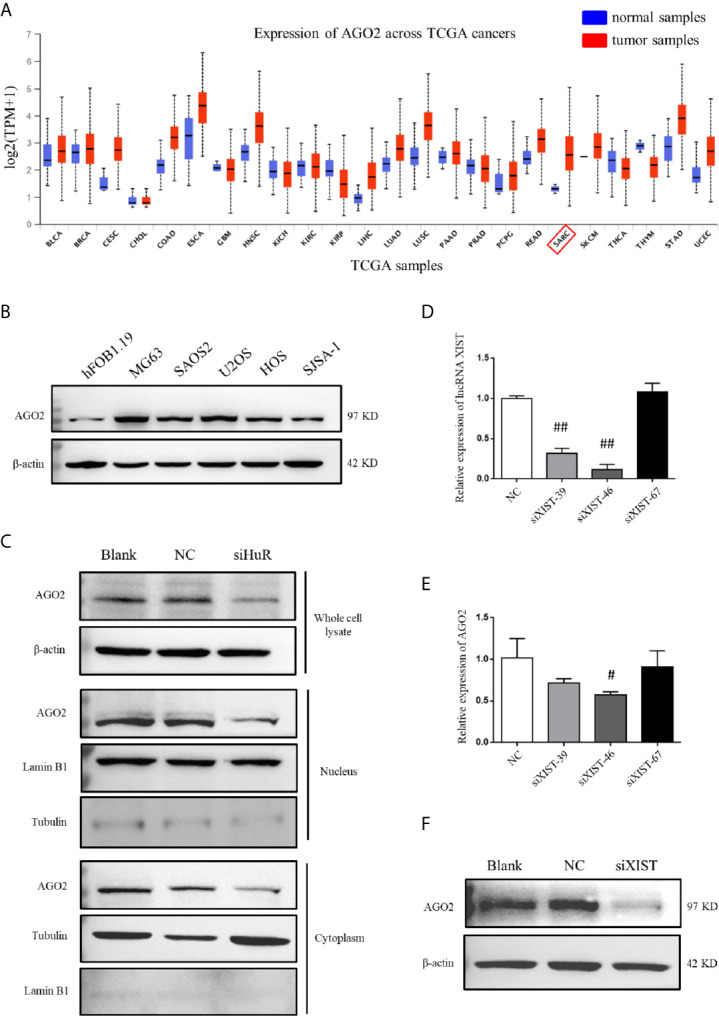
AGO2 expression was regulated by HuR and lncRNA XIST in OS cells. **(A)** Expression levels of AGO2 in multiple cancers and normal samples using data from TCGA database. SARC: sarcoma cancer. **(B)** Western blotting analysis of AGO2 expression in a human osteoblast cell line (hFOB1.19) and OS cell lines (MG63, SAOS2, U2OS, HOS, and SJSA-1). The blots in **Figure 1B** were stripped and re-probed for AGO2 protein. **(C)** Western blotting analysis of AGO2 expression in cell fractions after HuR knockdown. Whole cell lysate and nuclear and cytoplasmic fractions of cells with different treatment were isolated. Protein concentrations were measured by the Bradford assay, and equal amounts of protein were analyzed. β-actin, lamin B1, and tubulin served as loading controls for the whole cell lysate, nuclear fraction, and cytoplasmic fraction, respectively. **(D)** Expression of XIST in SJSA-1 cells transfected with NC siRNA or different siRNA against XIST was confirmed by qPCR analysis. ^##^ indicates *P* < 0.01 compared to the NC group. **(E)** mRNA levels of AGO2 in SJSA-1 cells transfected with NC siRNA or different siRNAs against XIST. ^#^ indicates *P* < 0.05 compared to the NC group. **(F)** Protein level of AGO2 in SJSA-1 cells transfected with NC siRNA or siRNA-46 against XIST and blank group without transfection.

Analysis by UALCAN showed that AGO2 was expressed at different levels in cases with multiple types of tumors, with a relatively large difference between normal tissues and tumors tissues in sarcoma cancer compared to other cancer types, indicating that a high AGO2 level is correlated with sarcoma progression (**Figure 5A**). AGO2 expression was determined in the OS cell lines MG63, SAOS2, U2OS, HOS, and SJSA-1, and normal hFOB1.19 cells. OS cells showed a significantly high level of AGO2 expression compared to that in normal osteoblast cells (**Figure 5B**). RISC is assembled by Dicer, argonaute protein, siRNA, and other biological macromolecules. Although AGO2-mediated RNA silencing mainly functions in the cytoplasm, western blotting revealed that knockdown of HuR diminished AGO2 protein levels both in the cell nucleus and cytoplasm (**Figure 5C**). Tubulin and lamin B1 levels were checked in the nucleus and cytoplasm to ensure that there was no contamination in the nucleus fraction and cytoplasm fraction.

To investigate the role of lncRNA XIST on AGO2, we designed three siRNAs specific to XIST. RNAi has been widely and effectively used to study lncRNA XIST deficiency (22, 23). qPCR demonstrated that siXIST-46 is the most effective siRNA for lncRNA XIST knockdown with an interference efficiency of over 80% (**Figure 5D**). qPCR and western blotting assays revealed that silencing of XIST significantly downregulated AGO2 at both mRNA and protein levels in OS cells (**Figures 5E, F**).

**Figure 6:**
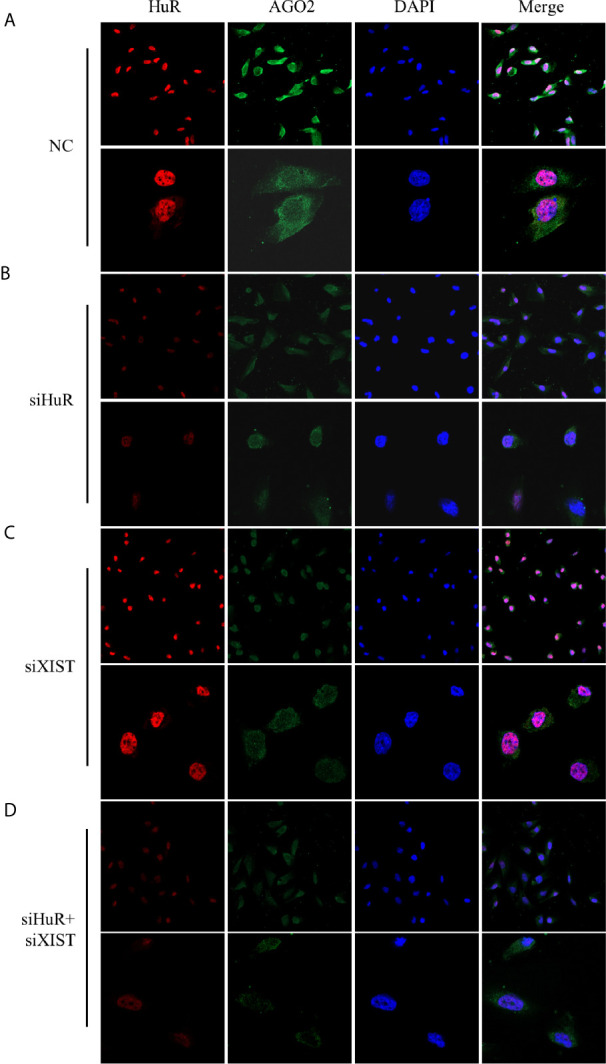
Confocal microscopic analysis of AGO2 expression in OS cells. Representative immunofluorescence analysis results for regulation of AGO2 by HuR and lncRNA XIST. SJSA-1 cells with different treatments were fixed with 1% paraformaldehyde and stained with anti-HuR (red) and anti-AGO2 (green) antibodies. Nuclei of cells appeared as blue because of DAPI nuclear staining. **(A)** Cells were transfected with an NC siRNA for 48 h. **(B)** Cells were transfected with a HuR siRNA for 48 h. **(C)** Cells were transfected with an lncRNA XIST siRNA for 48 h. **(D)** Cells were co-transfected with both HuR and lncRNA XIST siRNAs for 48 h. Stained cells were examined under a Leica confocal immunofluorescence microscope. Upper panel: original magnification 200x; lower panel, original magnification 630x.

Immunofluorescence staining and confocal analysis were conducted to observe the expression and subcellular localization of AGO2, which is regulated by HuR and lncRNA XIST. As shown in **Figure 6A**, most HuR was localized in the nucleus of SJSA-1 OS cells, whereas AGO2 was mainly distributed in the cytoplasm. siRNAs for both HuR and lncRNA XIST inhibited AGO2 expression; in contrast, siRNA for XIST did not influence the level and distribution of HuR (**Figures 6B, C**). The combination of HuR and lncRNA XIST siRNA strongly suppressed AGO2 protein expression (**Figure 6D**).

### AGO2 Is Involved in OS Cell Progression

**Figure 7:**
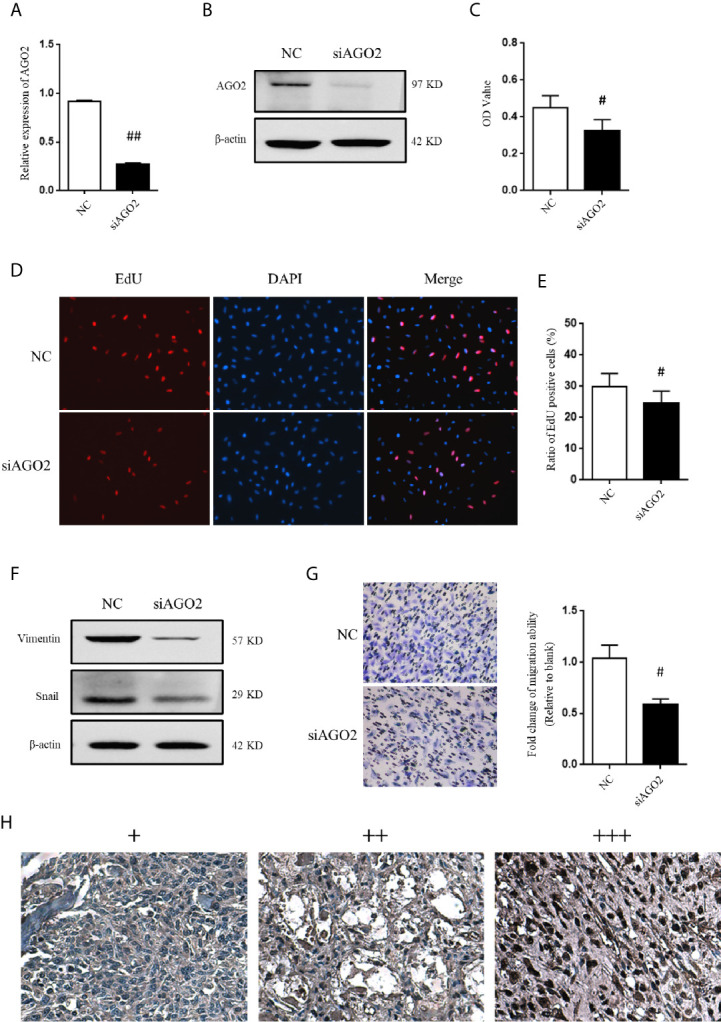
AGO2 regulates OS cell progression and migration. **(A)** qPCR and **(B)** western blotting analysis were performed to detect the efficiency of AGO2 siRNA. ^##^ indicates *P* < 0.01 compared to the NC group. **(C)** SJSA-1 cells with NC siRNA or AGO2 siRNA transfection were subjected to a CCK-8 assay to examine cell viability. ^#^ indicates *P* < 0.05 compared to the NC group. **(D)** EdU proliferation assay analysis was performed 48 h after AGO2 siRNA or NC siRNA transfection in SJSA-1 cells. Cell nucleus was stained with DAPI. Original magnification 200x. **(E)** Statistical graph of EdU assay. ^#^ indicates *P* < 0.05 compared to the NC group. **(F)** Representative images of western blotting for EMT markers vimentin and transcriptional factor Snail. β-actin served as an internal loading control. **(G)** Representative images of transwell migration assay of SJSA-1 cells with AGO2 knockdown and NC siRNA control. Statistical graph of transwell migration assay is shown. ^#^ indicates *P* < 0.05 compared to the NC group. Representative images from three separate experiments are shown. **(H)** Expression of AGO2 protein in OS tissues from patients with osteosarcoma. Representative images of different IHC staining intensities of AGO2 are shown in OS tissues. Staining patterns were categorized into three groups as follows: weak staining (+), moderate staining (++), and intense staining (+++). Original magnification 200x.

Considering that AGO2 was significantly regulated by HuR/lncRNA XIST, we further evaluated the role of AGO2 in OS cells. First, a specific siRNA effectively downregulated AGO2 expression at the mRNA and protein levels (**Figures 7A, B**). As shown in **Figure 7C**, the CCK-8 assay demonstrated that knockdown of AGO2 significantly inhibited OS cell viabilty compared to that in the NC siRNA control. The EdU incorporation assay for immunochemical detection of the nucleotide analog incorporated into replicated DNA showed consistent results with the CCK-8 assay (**Figures 7D, E**, *P* < 0.05).

Moreover, western blotting analysis indicated that silencing of AGO2 inhibited the level of the EMT-related molecule vimentin and Snail in OS cells (**Figure 7F**). Compared to cells transfected with NC siRNA, OS cells transfected with AGO2 siRNA exhibited lower levels of migration ability (**Figure 7G**) as detected in the transwell assay. Furthermore, we examined AGO2 expression in OS tissues by IHC staining. The results showed that AGO2 is localized in the both cell nucleus and cytoplasm (**Figure 7H**). Comparisons between AGO2 expression and the clinicopathological characteristics of OS are shown in **Table 2**. However, it was shown that AGO2 expression has no significant correlation with gender, age, and different stages of OS tumors. Taken together, these results suggest that HuR regulates OS cell tumorigenesis and progression by targeting AGO2 with the assistance of lncRNA XIST.

**Table 2:** Correlation between AGO2 and clinical features of patients with OS (n = 34).

Variable		No. of patients	AGO2 expression			*P*-values
			+	++	+++	
Gender	Male	22 (64.7)	10	4	8	0.483
	Female	12 (35.3)	7	3	2
Age	>20	24 (70.6)	12	4	8	0.596
	<20	10 (29.4)	5	3	2
T stage	T1	8 (23.5)	6	1	1	0.265
	T2	26 (76.5)	11	6	9

P-values based on a χ^2^ test.

## Discussion

Overexpression of HuR occurs during tumorigenesis and cancer progression in various cancer types (9). Thus, inhibition of HuR biological function is an attractive goal in cancer research. Recently, the HuR small molecule inhibitor MS-444 was developed and shown to attenuate the invasion of glioblastoma cells and growth of colorectal cancer cells (20, 24). The CMLD-2 inhibitor exhibited antitumor effects *via* MAD2 in thyroid cancer cells (25).

Recently, Pan et al. showed that knockdown of HuR inhibited MG63 OS cell viability and EMT and promoted cell apoptosis by suppressing the miR-142-3p and high mobility group AT-hook 1 axis (12). Xu et al. demonstrated that HuR suppresses OS cell migration, invasion, and EMT by inhibiting YAP activation, which is a key executor in the Hippo signaling pathway (11). These studies demonstrate that HuR expression was significantly increased in OS tissues compared to that in adjacent normal tissues. Here, we found that HuR expression significantly differed between the T1 and T2 stages of OS tumors. In this study, both the IHC staining and immunofluorescence assays showed that HuR is mainly localized in the OS cell nucleus. This is not consistent with the consensus that HuR is mostly localized in the nucleus of normal cells, but typically translocated to the cytoplasmic region of malignant cells (9). An increased nuclear HuR expression pattern was more frequently observed in invasive epithelial tumors than that in low malignant potential ovarian tumors (26). However, according to this observation, some molecules likely help HuR to carry out its function in OS cells.

Aberrant expression of lncRNAs has been detected in multiple cancers and may be useful as diagnostic and prognostic markers in cancer progression (27). Moreover, lncRNAs have been investigated as key modulators that regulate many biological processes in human cancers *via* diverse mechanisms and may act as decoys, scaffolds, and enhancer RNAs (28). LncRNA was reported to regulate gene expression and function by cooperating with HuR in different manners, such as sponges or by recruiting HuR/miRNA, competitively/blocking binding to HuR, and stabilizing HuR protein (29–31).

LncRNA XIST expression was significantly upregulated in OS tissues, and high XIST expression is associated with tumor size, advanced clinical stage, and distant metastasis (17, 21). The mechanisms have been investigated previously, including the involvement of XIST in NK-κB/PUMA signaling (32), repression of P21 expression to regulate the cell cycle by binding EZH2 (33), targeting of YAP *via* miR-195-5p (34), regulation of miR-21-5p/PDCD4 or miR-193a-3p (35, 36), or sponging of miRNA-137 (37). In the current study, we demonstrated that XIST directly binds to the HuR protein and is regulated by HuR in OS cells, suggesting that XIST is associated with HuR-mediated OS tumor progression.

Furthermore, HuR may stabilize target mRNAs and increase the translation of numerous mRNAs while repressing the translation of other mRNAs (e.g., IGF-ІR). Therefore, we investigated the target modulated by HuR in OS cells. As an important enzyme in the RNA interference pathway, AGO2 is differentially expressed in many tumors and is related to the clinical stage and pathological grading (38, 39). However, our results showed no significant correlation between AGO2 expression and typical clinical characteristics (like gender, age, and stages) of OS tumors, which may result from the limited sample amount of OS cases, so the studies of larger sample sizes are needed to verify these findings. AGO2 protein is widely expressed in organisms; as a regulatory element, it may play a role in inhibiting or promoting tumors, depending on the miRNA to which it binds. Kim et al. reported that HuR is associated with AGO2 in an RNA-dependent manner according to co-IP analyses, and HuR is necessary for the AGO2/let7 interaction with *c-Myc* mRNA in HeLa cells (40). Here, we found that HuR bound to AGO2, and further demonstrated that lncRNA XIST may function as a mediator of HuR-induced AGO2 modulation. Thus, the inhibition effect of AGO2 on OS migration and EMT by downregulating lncRNA XIST expression is a new function for the RNA-binding protein HuR.

In conclusion, mechanistic studies indicated that downregulation of HuR significantly inhibited OS cell migration and EMT by regulating the lncRNA XIST/AGO2 signaling pathway.

## Data Availability Statement

The raw data supporting the conclusions of this article will be made available by the authors, without undue reservation.

## Ethics Statement

Ethical approval was not required for the commercialized OS tissue array samples in accordance with the institutional requirements.

## Author Contributions

YD, YZho, RZ designed the study. YL, YZha, JZ performed the *in vitro* experiments. JM, XX, and YW prepared the figures. ZZ, DJ, and SS collected and analyzed the data. YZho, RZ wrote the manuscript. All authors contributed to the article and approved the submitted version.

## Funding

This investigation was supported by the Natural Science Foundation of China (No. 81871258 and No. 81671575).

## Conflict of Interest

The authors declare that the research was conducted in the absence of any commercial or financial relationships that could be construed as a potential conflict of interest.

## Supplementary Material

Supplementary Figure 1 Effect of HuR overexpression on apoptosis of OS cells. **(A)** Representative image of western blotting for HuR and Bcl-2. β-actin served as an internal loading control. **(B)** TUNEL staining in the SJSA-1 cells harvested from the HuR overexpression (OE) group and HuR siRNA + OE group. Scale bar = 125 μm.

## Abbreviations

Ago, Argonaute; AREs, AU-rich elements; CCK-8, cell counting kit-8; DAPI, 4',6-diamidino-2-phenylindole; EdU, 5-ethynyl-2'-deoxyuridine; ELAVL1, ELAV like RNA-binding protein 1; EMT, epithelial-mesenchymal transition; FISH, fluorescence in situ hybridization; HuR, human antigen R; IHC, immunohistochemistry; IP, immunoprecipitation; lncRNA, long noncoding RNA; OS, osteosarcoma; PBS, phosphate-buffered saline; qPCR, quantitative real-time PCR; RISC, RNA-induced silencing complex; siRNA, small interfering RNA; XIST, X-inactive specific transcript.
