# Supplementary figures and images for: Silencing of HuR Inhibits Osteosarcoma Cell Epithelial-Mesenchymal Transition via AGO2 in Association With Long Non-Coding RNA XIST
Source: Front Oncol. 2021 Mar 19;11:601982. doi: 10.3389/fonc.2021.601982 (PMC8017292; doi:10.3389/fonc.2021.601982)

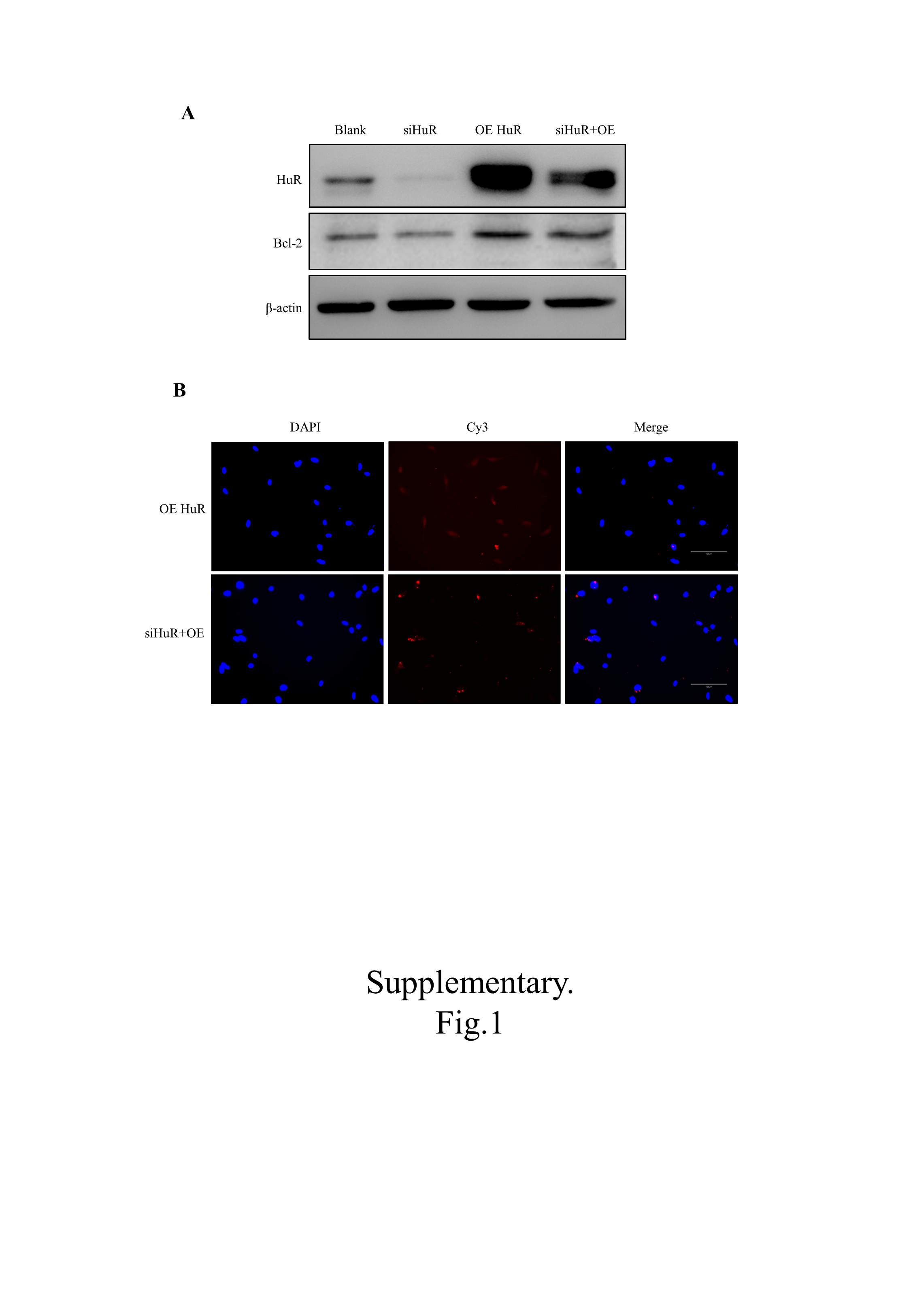

Supplement: Supplementary Figure 1 — Effect of HuR overexpression on apoptosis of OS cells. (A) Representative image of western blotting for HuR and Bcl-2. β-actin served as an internal loading control. (B) TUNEL staining in the SJSA-1 cells harvested from the HuR overexpression (OE) group and HuR siRNA + OE group. Scale bar = 125 μm. [file Image_1.tif]
